# Evidence of Effect of Aerobic Exercise on Cognitive Intervention in Older Adults With Mild Cognitive Impairment

**DOI:** 10.3389/fpsyt.2021.713671

**Published:** 2021-07-20

**Authors:** Liming Yong, Lei Liu, Ting Ding, Gao Yang, Haibing Su, Jibing Wang, Ming Yang, Jindong Chang

**Affiliations:** ^1^School of Physical Education, Southwest University, Chongqing, China; ^2^Institute of Motor Quotient, Southwest University, Chongqing, China; ^3^Qingdao Mental Health Center, Qingdao University, Qingdao, China; ^4^International College of Football, Tongji University, Shanghai, China

**Keywords:** aerobic exercise, mild cognitive impairment, RCTs, systematic review, meta-analysis

## Abstract

This study aimed to evaluate the effectiveness of aerobic exercise as a cognitive intervention for older adults with mild cognitive impairment (MCI). The PubMed, EMBASE (Ovid), Cochrane Library, Web of Science, and Medline databases were searched from their inception until 30 April 2021. Randomized controlled trials (RCTs) examining the effects of aerobic exercise on global cognitive function in older adults with MCI were included. Ten eligible trials with acceptable methodological quality were identified. The meta-analysis results showed that aerobic exercise significantly improved the MMSE (*N* = 956, *MD* = 0.60, 95% CI: 0.28–0.92, *p* = 0.0003, *I*^2^ = 31%, fixed effects model) and MoCA scores (*N* = 398, *MD* = 1.67, 95% CI. 1.18–2.15, *p* < 0.0001, *I*^2^ = 37%, fixed-effects model) and overall cognitive performance in patients with MCI. The results of this study suggest that participation in regular aerobic exercise can improve cognitive function in older adults with MCI. These findings should be used with caution considering the limitations of the study.

## Introduction

Mild cognitive impairment (MCI) is a typical transitional stage between the normal aging process and the onset of dementia. Mild cognitive impairment includes a group of cognitive impairment conditions presenting in the early stages of dementia (1). According to recent studies involving people over 60 years of age, the prevalence of MCI is about 15% (2, 3), while another cohort study in Beijing, China showed that a prevalence of MCI in older adults of 16.6% (4). In addition, domestic studies have reported conversion rates from MCI to dementia and Alzheimer's disease (AD) of 34 and 28%, respectively (2, 3). There is no positive evidence to support the use of pharmacological interventions to attenuate cognitive decline in MCI patients (5–8). Instead, risk reduction factors (9) and participation in physical activity are widely considered to be effective non-pharmacological interventions. Although studies have investigated the effects of non-pharmacological interventions including diet (10), social relationships (7, 11), and cognitive training (12–14), the advantages of exercise/physical activity are unknown.

A growing number of studies are suggesting that physical activity/exercise may improve cognitive function. However, a meta-analysis of 14 randomized controlled trials (RCTs) reported that there is no significant evidence that physical activity/exercise improves cognitive function in patients with MCI (15). However, in an intervention involving individuals with MCI, the Hamer prospective study review demonstrated an inverse relationship between physical activity/exercise and the risk of cognitive decline in healthy older adults (14). This evidence is also supported by the results of cross-sectional studies, longitudinal observational studies, and prospective intervention trials (16–21).

Several studies have investigated with beneficial effects of aerobic exercise in older adults with MCI in order to better understand the effects of exercise and how these change depending on the type of exercise performed (8, 15, 16). Although 11 previous aerobic exercise meta-analysis studies found favorable effects on overall cognitive performance and memory in older adults, the effects of beneficial effects on older adults with MCI varied widely depending on the type, frequency, and duration of aerobic exercise performed. Furthermore, it has been suggested that due to differences in measurement instruments used, results regarding the effects of aerobic exercise on cognitive interventions in older adults with MCI have not been uniform (15). Therefore, the purpose of this systematic evaluation and meta-analysis was to investigate the effects of exercise interventions on older adults with MCI based on the measurement tools used and the type of exercise performed and to evaluate the exercise intervention modality recommended in a RCT.

## Methods

### Search Strategy

This study used the Preferred Reporting Items for Systematic Reviews and Meta-Analyses guidelines (22). We searched for relevant studies written in English in PubMed, EMBASE (Ovid), Cochrane Library, Web of Science, Medline, and other databases for aerobics-related subject terms (e.g., aerobic exercise, aerobic dance, Qigong, Tai Chi, Yoga, physical activity) and cognitive impairment-related subject headings (e.g., MCI, memory loss, cognitive impairment). All search terms were grouped together as much as possible to find all relevant studies. In addition, previously cited studies were manually added if they met the inclusion criteria. Supplementary Materials used for the search strategy of the articles were available online (See Appendix 1. The literature was searched from its inception to April 30, 2021).

### Inclusion and Exclusion Criteria

The following inclusion criteria had to be met for exercise intervention trials to be included in this review: (1) Study design: RCTs, including those published by peer review or peer-reviewed journals in print; (2) Participants: older adults (60 years and older) with MCI that met the existing diagnostic criteria. Those with cognitive impairment or other neurological impairments due to AD or dementia were excluded from the study; (3) Intervention: the experimental group performed different types of aerobic exercise (e.g., yoga, tai chi, running, walking, dancing, etc.) for at least 8 weeks and exercised at least once per week; (4) Control: the control group did not perform specific exercise interventions, i.e., the control group only maintained their usual physical activity or performed sham exercises (e.g., stretching and balancing, exercise education, etc.); (5) Outcome: the subjects' overall cognitive abilities or specific cognitive domain abilities such as memory, attention, etc. were measured by any of the Mini-Mental State Examination (MMSE) and Montreal Cognitive Assessment (MoCA) measurement instruments. Studies without available data were excluded.

### Assessing the Risk of Bias

The Cochrane Risk of Bias tool was used to assess the risk of bias in the included trials (23). In addition, selection bias, performance bias, detection bias, attrition bias, reporting bias, and other types of bias were assessed, and each study was categorized as high, low, or unclear based on the degree of bias present. This work was done independently by two evaluators (LY and GY). Disagreements between the two reviewers were resolved through discussion with the two corresponding authors (JW and JC).

### Statistical Analysis

RevMan version 5.4.1 software (Cochrane) was used for this meta-analysis of the study. The mean difference (MD) or standardized difference (SMD) statistic was used to calculate the combined effect size, and the corresponding 95% CI was used to summarize the data. If the fit heterogeneity of the selected study data was small (*I*^2^ ≤ 50%, *p* > 0.05), the analysis model was deemed to be a fixed-effects model. If the data heterogeneity of the selected studies was high (*I*^2^ > 50%, *p* < 0.05), the analytical model was deemed to be a random-effects model. However, when the level of heterogeneity between studies was high (*I*^2^> 75%), the overall combined analysis was considered inappropriate due to reasons of heterogeneity, including the characteristics of the measurement instruments. Statistical heterogeneity in the included studies was assessed using the χ^2^-test and *I*^2^-values, with *I*^2^ > 75% indicating a high degree of statistical heterogeneity (24).

## Results

### Study Identification

**Figure 1:**
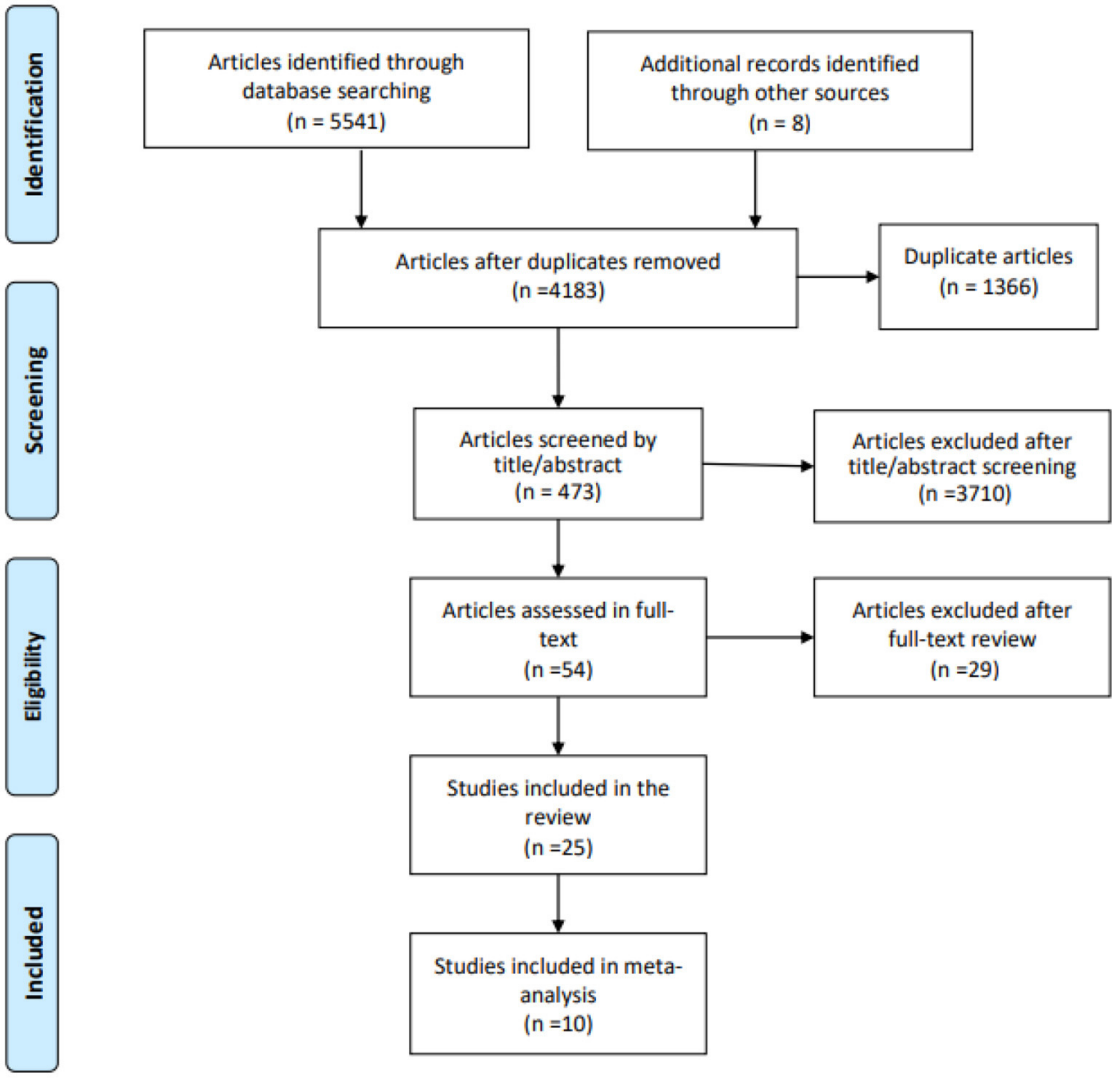
Flowchart of the selected literature.

Following a predetermined search strategy, 5,541 records were initially identified from four major electronic databases. Two reviewers (LY and GY) removed 5,487 irrelevant studies based on their abstracts and titles, leaving a total of 54 potential studies to be further assessed for eligibility. Following assessment, 10 studies were included in a systematic review and meta-analysis involving 1,364 MCI participants. Figure 1 shows the detailed process used to screen eligible studies.

### Characteristics of the Included Studies

Table 1 presents the characteristics of each included study. A total of 10 RCTs involving 1,364 MCI participants (454 men and 910 women, mean age 73.0 ± 6.72 years) were included in the analysis. All studies were published between 2012 and 2021. The study countries and regions included China (26, 31, 34) (*n* = 3), Hong Kong (28, 29) (*n* = 2), Japan (32) (*n* = 1), Pakistan (25) (*n* = 1), Greece (30) (*n* = 1), Denmark (27) (*n* = 1), and Spain (33) (*n* = 1). All studies were conducted in Asian and European countries, and most of the studies were conducted in developed countries or regions. The majority of the study participants were recruited from the community or from nursing homes. All included studies clearly reported the criteria used for participant enrollment.

**Table 1:** Characteristics of included trials in this review.

**Author, year (Ref.)**	**N(m/f)**	**Age**	**Exercise program**	**Design**	**Cognitive test**	**Cognitive domain**	**Exercise stimulus**	**Control condition**
Amjad, 2019 (25) (Pakistan)	40(21/19)	CG: 59.56 ± 2.65 EG: 58.23 ± 2.31	EG: treadmill, stationary bicycle, (aerobic exercise) CG: stretch	RCT	MoCA	Global cognitive function	6 weeks, 3 d/wk, 20–40 min/d, 60–80% of maximum heart rate	Stretch
Chang, 2021 (26) (China)	109(0/109)	CG: 75.94 ± 3.61 EG: 75.56 ± 3.60	EG: square dance CG: daily lifestyle	Cluster-RCT	MoCA	Global cognitive function	18 weeks, 3 d/wk, 30 min/d, heart rate 100–140 beats/min	Daily lifestyle
Hoffmann, 2015 (27) (Danmark)	200(113/87)	CG: 71.3 ± 7.3 EG: 69.8 ±7.4	EG: walking CG: received treatment as usual	RCT	MMSE	Global cognitive function Immediate/ Delayed recall	16 weeks, 3 d/wk, 60 min/d	Received treatment as usual
Lam, 2012 (28) (Hong Kong, China)	261(92/169)	CG:78.3 ± 6.6 EG:77.2 ± 6.3	EG: Tai Chi CG: stretching and toning exercise	RCT	MMSE	Global cognitive function Delayed recall, attention, executive function, verbal fluency	1 year, 3 d/wk, 30 min/d, moderate intensity	Stretching and toning exercise
Lam, 2015 (29) (Hong Kong, China)	263(54/206)	CG:75.4 ± 6.1 EG:76.3 ± 6.6	EG: Tai Chi, static bicycle riding and other physical activity CG: social activity	RCT	MMSE	Global cognitive function Delayed recall Verbal fluency	1 year, 1 d/wk, 60 min/d, moderate intensity	Stretching and toning exercise
Lazarou, 2017 (30) (Greece)	129(28/101)	CG:67.92 ± 9.47 EG:65.89 ± 10.76	EG: Dance CG: usual physical activity	RCT	MoCA	Global cognitive function	10 months, 2/wk, 60 min/d	No intervention
Song, 2019 (31) (China)	120(30/90)	CG: 75.33 ± 6.78 EG: 76.22 ± 5.76	EG: aerobic stepping exercise CG: education program	RCT	MoCA	Global cognitive function	16 weeks, 3 d/wk, 60 min exercise/d	8 biweekly 45 min education programs
Doi, 2017 (32) (Japan)	134(52/82)	CG:76 ± 4.9 EG:75.7 ± 4.1	EG: Dance CG: usual physical activity	RCT	MMSE	Global cognitive function	40 weeks, 60 min/wk	40-week 3 health education classes (90 min/class)
Varela, 2012 (33) (Spain)	48(21/27)	EG: 77.88 ± 10.71 CG: 79.40 ± 6.72	EG: cycling CG: recreational activity	RCT	MMSE	Global cognitive function	3 months, 3 d/wk, 30 min/d, 40–60% maximum heart rate	Recreational activities (playing cards, reading newspaper)
Wei, 2014 (34) (China)	60(40/20)	CG:65.27 ± 4.63 EG:66.73 ± 5.48	EG: handball training CG: entertainment	RCT	MMSE	Global cognitive function	6 months, 5 d/wk,30 min/d, 60%Hrmax	Traditional life entertainment (playing cards, etc.)

The aerobic exercise modalities included in the studies were diverse. There were three dance-based studies (26, 30, 32), two mixed-modality studies (Tai Chi, treadmill, stationary bike, or other) (25, 29), one walking studies (27), one Tai Chi study (28), one aerobic stepping study (31), one cycling study (33), and one handball training study (34). The duration of aerobic exercise interventions was mostly 2–3 times per week for 20–60 min, except for the studies conducted by Lam et al. (28) and Wei et al. (34), which used one and five times, respectively. The intervention duration ranged from 4 months to 1 year, except for one study that used 6 weeks (25). Three of the ten studies compared aerobic exercise with no intervention (i.e., regular physical activity) (26, 27, 30), three studies compared it with stretching activities (25, 28, 29), two studies compared it with educational activities (31, 32), and two studies compared it with recreational activities (33, 34). Even though these studies compared aerobic exercise with stretching activities, educational activities, or recreational activities, these activities were not different from regular physical activity due to their lower level of intensity. They did not change participants' exercise behavior. In the four included studies, during exercise, heart rate was controlled at 40–60% (33, 34) or 60–80% of the participants' maximum (25) or between 100 and 140 (26) to ensure an aerobic level of intensity was maintained. Other heart rates were described as moderate intensity. Two different cognitive assessment tools, MMSE and MoCA, were used to assess the same cognitive domain within or among studies.

### Bias Risk of the Included Studies

**Figure 2:**
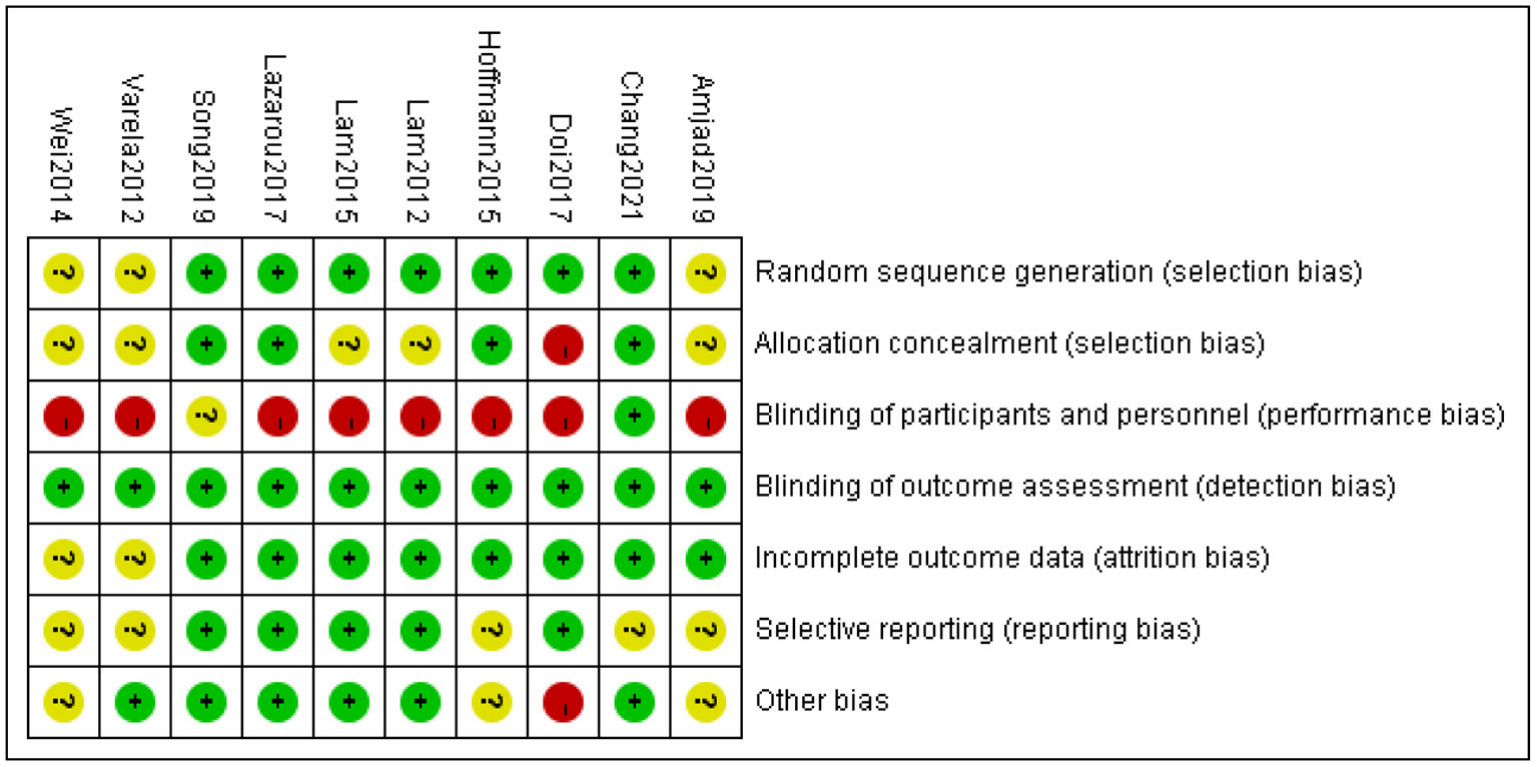
Risk of bias summary of inclusion studies.

Figure 2 summarizes the risk of bias for the included studies. All included trials used the random assignment method, with seven of them using the method of random number generation of random sequences. However, there were three studies with unclear random assignment methods. The subject selection bias was unclear in five studies, and it was high risk in one study. Only one study reported the use of specific allocation concealment through envelope preservation (31). The subject selection bias of five studies was unclear, and for one study, it was high risk. In all included studies, the risk of potential performance bias was high because it was difficult to blind participants to the exercise intervention. Only one cluster-randomized trial was consistent with this potentially performance-biased blinded design (26). All included studies blinded the outcome assessors; therefore, their risk of detection bias was judged as low. The risk of completeness bias for two studies was unclear based on the completeness of the study data or the number of studies describing missing data. The risk of selective reporting bias was judged to be low in five studies after examining the available protocols. The risk of other types of bias was high for one study and unclear for three studies due to their limited sample sizes and unclear comparison with baseline characteristics.

### Effect of Intervention

**Figure 3:**
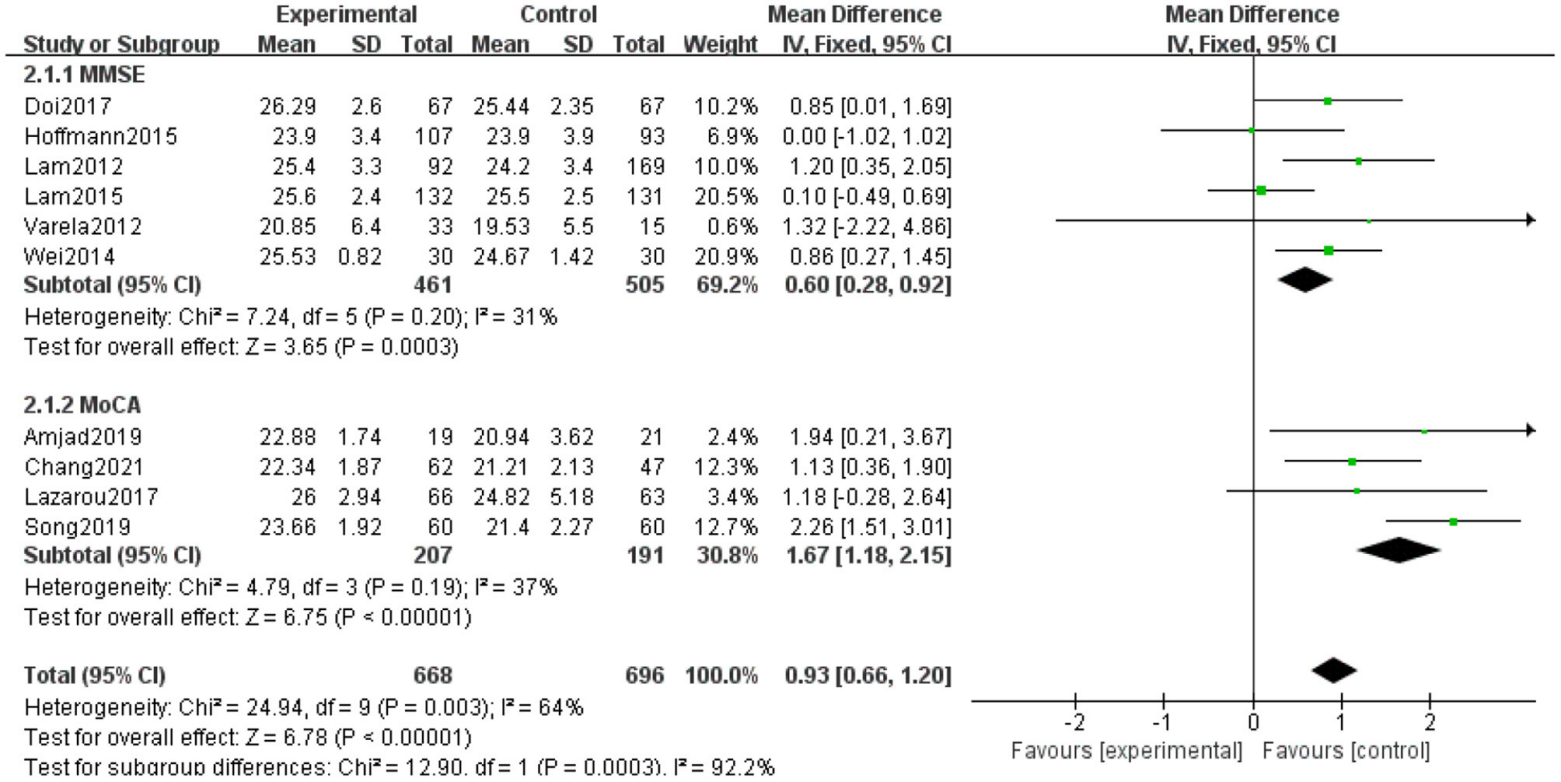
Forest plot for aerobic exercise on global cognition function with MCI older. MMSE, Mini-Mental State Examination; MoCA, Montreal Cognitive Assessment.

The effect of aerobic exercise on the overall cognitive performance of MCI patients was tested by assessing cognitive function using the MMSE in six of the ten global impact studies and the MoCA in four studies. The combined analysis of the findings obtained by these two measurement tools was inappropriate due to the inconsistent results between studies that used different measurement tools. Therefore, an analysis of subgroups was required. The results of the subgroup analysis showed that aerobic exercise significantly improves the overall cognitive performance of patients with MCI as shown by significant improvements in MMSE (*N* = 956, *MD* = 0.60, 95% CI: 0.28–0.92, *p* = 0.0003, *I*^2^ = 31%, fixed-effects model) and MoCA scores (*N* = 398, *MD* = 1.67, 95% CI: 1.18–2.15, *p* < 0.0001, *I*^2^ = 37%), significantly improved overall cognitive performance in patients with MCI (Figure 3).

## Discussion

This systematic review and meta-analysis provides positive evidence that aerobic exercise significantly improves cognitive performance in older adults with MCI.

None of the included studies reported adverse events related to aerobic exercise. However, the results of some other studies are inconsistent with ours. The use of different trial periods may have led to inconsistency in results. For example, some reports showed no difference between the experimental and control groups in the area of overall cognitive function following a 3-month intervention (35). In contrast, other trials with longer intervention or follow-up periods identified an improvement in cognitive function (29, 30, 32, 36). In addition, a study using the MoCA measurement tool found no significant improvement after 3 months of intervention (36), while another study showed significant effects after a 10-month intervention (30). Only four of the included studies specified aerobic exercise intensity with target peak levels of 40–60%, 60–80%, 60%, and a heart rate maintained at 100–140 beats per minute (25, 26, 33, 34). This indicates that exercise intensity may be a factor associated with inconsistent results.

A review study proposed that location and gender may affect the impact of an intervention. In this study, three studies were conducted in mainland China, two studies were from Hong Kong, China, and the remaining studies were conducted in Japan, Pakistan, Denmark, Spain, and Greece. Geographically, six studies were conducted in East Asia (26, 28, 29, 31, 32, 34) and four were conducted in Europe and South Asia (25, 27, 30, 33). Geographical concentration may influence the effect of an intervention. In addition, the influence of the gender factor is mainly reflected in the balanced distribution of subjects. For example, in Chang et al.'s study (26, 37), all participants were female, so their study design excluded the interference of gender factors in the experiment. In contrast, in the study designs of Lam et al. (28) and Lazarous et al. (30), the proportion of females was more than 75%, which implies a three-to-one gap.

## Strengths and Limitations

The strength of this review lies in its systematic approach design. The strength of our design is reflected in three main areas. First, we focused on aerobic exercise. By defining the concept of aerobic exercise, the use of moderate-intensity exercise was identified as an inclusion criterion. In addition, the selection of exercise programs for MCI patients included tai chi, qigong, dance (square dance), yoga, running, walking, and stepping. Second, we focused on the use of measurement tools. Only trials measured by two instruments, MMSE and MoCA, were included in the study. This was done to allow us to validate the consistency of the results and to prevent the inclusion of multiple measurement tools from affecting the validity of the analysis. Third, we only discussed the effects of aerobic exercise on global cognitive function in MCI patients and not on specific domains of cognitive function. This allowed us to focus more on the effects of the intervention and thus identify whether aerobic exercise can affect the cognitive performance of patients with MCI.

This study has several limitations. First, the types of aerobic exercise included in the study were diverse, and it is uncertain whether consistent effects were observed due to the different exercise programs used. Second, the frequency and intensity of exercise varied among studies, and the resulting mechanisms of exercise are unclear. Third, although the two measurement tools used have high reliability and validity, their objective measurements need to be further enhanced, for example, through the use of EEG or MRI. Fourth, the standardization of RCTs still needs to be improved. Fifth, insufficient sample size may have seriously limited the interpretability of the results, with three of the 10 studies having sample sizes of <100 individuals. In conclusion, the results of this study confirm the positive effect of aerobic exercise as an intervention for MCI patients. However, the above deficiencies remind us that the effect values provided by the meta-analysis should be interpreted with caution.

## Conclusion

Participation in aerobic exercise can contribute to the improvement of overall cognitive function in elderly MCI patients. However, the mechanisms by which aerobic exercise influences the overall cognition of MCI patients are unclear due to differences in the type, frequency, and duration of aerobic exercise used in previous studies. Large-scale, rigorous RCTs are needed to explore specific areas of impact and mechanisms of action and determine appropriate intervention programs.

## Data Availability Statement

The raw data supporting the conclusions of this article will be made available by the authors, without undue reservation.

## Author Contributions

LY, GY, and HS: data collection. LY, LL, TD, JC, and MY: data analysis, conception, and design. LY, JW, JC, and MY: research design, writing the manuscript, and revision. All authors contributed to the article and approved the submitted version.

## Conflict of Interest

The authors declare that the research was conducted in the absence of any commercial or financial relationships that could be construed as a potential conflict of interest.
