# Supplementary material for: Evidence of Effect of Aerobic Exercise on Cognitive Intervention in Older Adults With Mild Cognitive Impairment
Source: Front Psychiatry. 2021 Jul 20;12:713671. doi: 10.3389/fpsyt.2021.713671 (PMC8329556; doi:10.3389/fpsyt.2021.713671)
Supplement: Supplementary file 1 [file Presentation_1.pdf]

Search terms

(((aerobic exercise [Abstract]) OR (dancing [Abstract]) OR (Qigong[Abstract]) OR (Tai chi[Abstract]) OR (Yoga [Abstract]) OR (physical activity [Abstract]) OR (physical exercise [Abstract])) AND ((cognitive impairment[Abstract]) OR (mild cognitive impairment[Abstract]) OR (MCI[Abstract]) OR (memory impairment [Abstract]) OR (memory loss[Abstract])) AND ((older[Abstract]) OR(elderly [Abstract])))

(((aerobic exercise [MeSH Terms]) OR (dancing [MeSH Terms]) OR (Qigong[MeSH Terms]) OR (Tai chi[MeSH Terms]) OR (Yoga [MeSH Terms]) OR (physical activity [MeSH Terms]) OR (physical exercise [MeSH Terms])) AND ((cognitive impairment[MeSH Terms]) OR (mild cognitive impairment[MeSH Terms]) OR (MCI[MeSH Terms]) OR (memory impairment [MeSH Terms]) OR (memory loss[MeSH Terms])) AND ((older[MeSH Terms]) OR(elderly [MeSH Terms])))

(((aerobic exercise [Title]) OR (dancing [Title]) OR (Qigong[Title]) OR (Tai chi[Title]) OR (Yoga [Title]) OR (physical activity [Title]) OR (physical exercise [Title])) AND ((cognitive impairment[Title]) OR (mild cognitive impairment[Title]) OR (MCI[Title]) OR (memory impairment [Title]) OR (memory loss[Title])) AND ((older[Title]) OR(elderly [Title])))
